# Supplementary material for: Protease-Triggered Release of Stabilized CXCL12 from Coated Scaffolds in an Ex Vivo Wound Model
Source: Pharmaceutics. 2021 Oct 1;13(10):1597. doi: 10.3390/pharmaceutics13101597 (PMC8539926; doi:10.3390/pharmaceutics13101597)
Supplement: Supplementary file 1 [file pharmaceutics-13-01597-s001.zip › pharmaceutics-1377013-supplementary.pdf]

pharmaceutics
MDPI

Supplementary Materials: Protease-Triggered Release of Stabilized CXCL12 From Coated Scaffolds in an Ex Vivo Wound Model

Sabrina Spiller, Tom Wippold, Kathrin Bellmann-Sickert, Sandra Franz, Anja Saalbach, Ulf Anderegg and Annette G. Beck-Sickinger

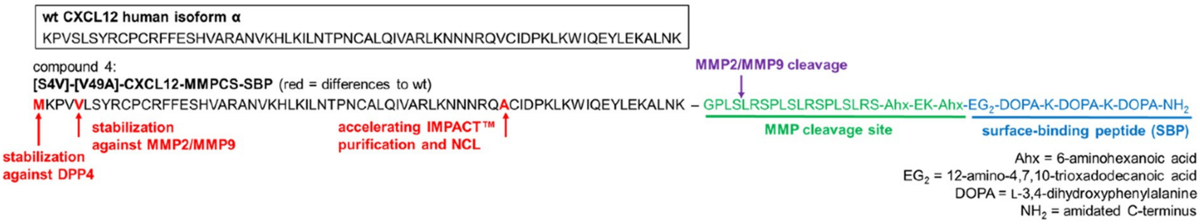

Figure S1. Sequence of wild type CXCL12 as well as introduced modifications and properties of compound 4.

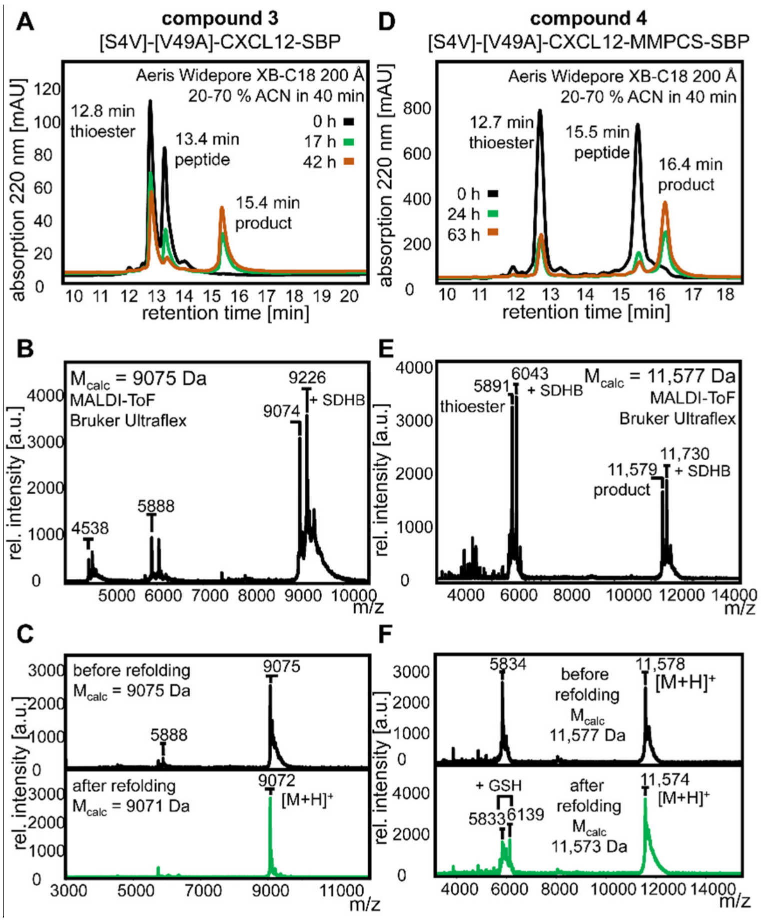

Figure S2. Analytics of NCL and refolding exemplarily for compound 3 (left) and compound 4 (right). (**A+D**) NCL was monitored during reaction by analytical RP-HPLC on a Phenomenex Aeris Widepore XB-C18 column (200 Å) using linear gradients of 0.1% TFA/H_2O and 0.08% TFA/ACN as indicated. (**B+E**) Identity of the product after NCL was proven by MALDI-ToF MS. (**C+F**) Successful refolding was verified by MALDI-ToF MS, showing a mass difference of *m*/*z* -4. GSH = glutathione, SDHB = super-dihydroxybenzoic acid matrix substance (*m*/*z* +152), M_{calc} = calculated molecular weight, ACN = acetonitrile.

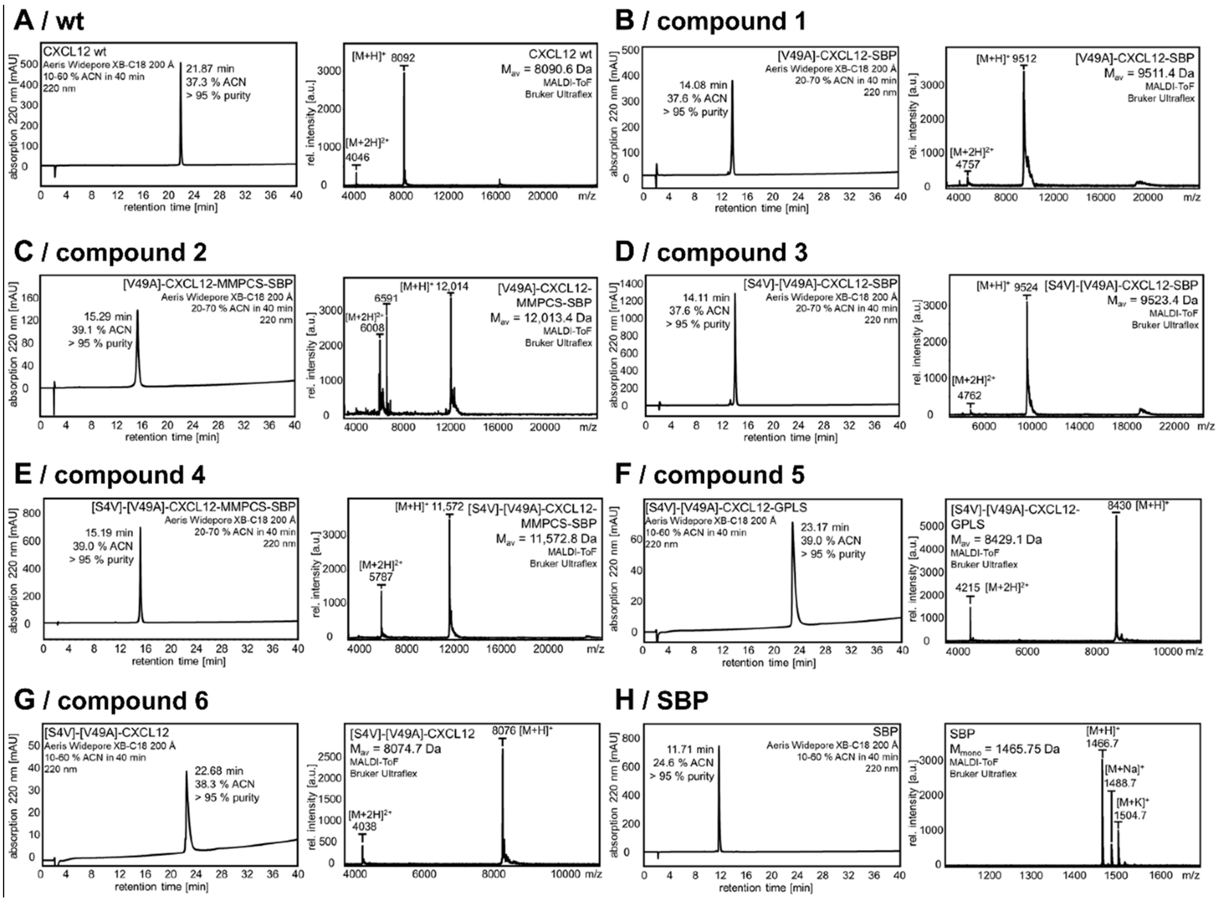

Figure S3. Analytics of generated compounds. Analytical RP-HPLC (left) was done on a Phenomenex Aeris Widepore XB-C18 column (200 Å) using linear gradients of 0.1% TFA/H2O and 0.08% TFA/ACN as indicated. Identity was proven by MALDI-ToF MS (right). Analysis was done for (**A**) CXCL12 wt, (**B**) compound 1 ([V49A]-CXCL12-SBP(Biotin), (**C**) compound 2 ([V49A]-CXCL12-MMPCS-SBP(Biotin), (**D**) compound 3 ([S4V]-[V49A]-CXCL12-SBP, (**E**) compound 4 ([S4V]-[V49A]-CXCL12-MMPCS-SBP, (**F**) compound 5 ([S4V]-[V49A]-CXCL12-GPLS), (**G**) compound 6 ([S4V]-[V49A]-CXCL12), and (**H**) SBP. wt = wild type, SBP = surface-binding peptide, M_{av} = calculated average molecular weight, M_{mono} = calculated monoisotopic molecular weight, ACN = acetonitrile.

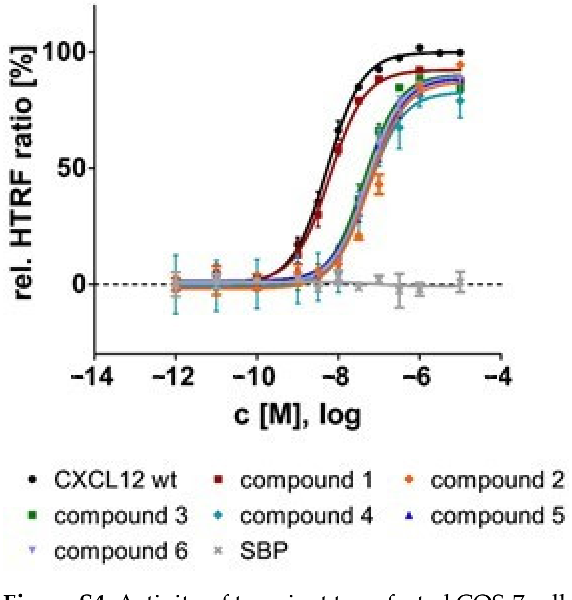

Figure S4. Activity of transient transfected COS-7 cells with the CXCR4 and a chimeric Gαqi protein after stimulation with CXCL12 variants for 1 h. IP accumulation was detected by FRET-donor/acceptor-labeled antibodies and homogenous time resolved fluorescence (HTRF) was measured. Data was normalized to CXCL12 wt and is presented as mean ± SEM, $n \geq 3$.

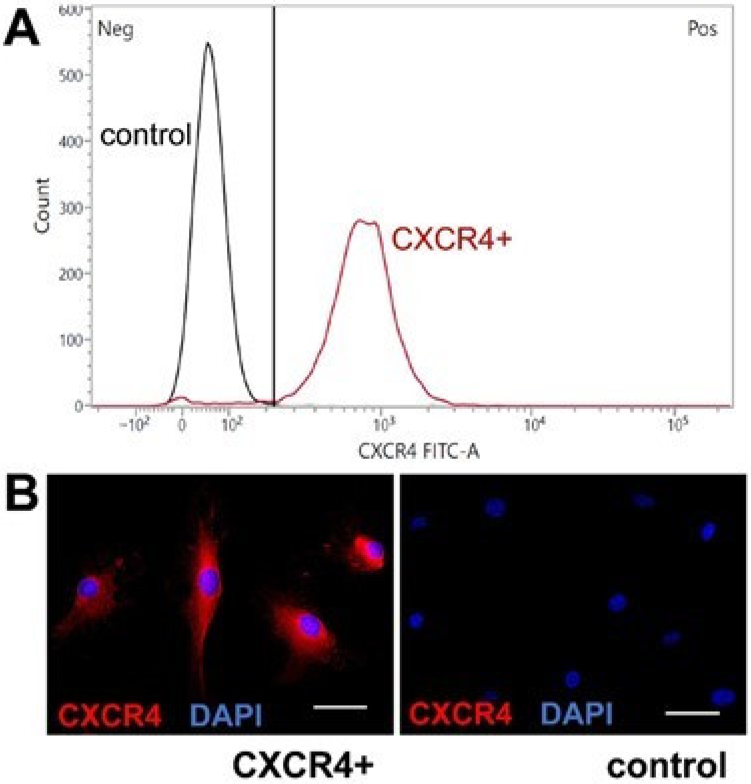

Figure S5. Characterization of murine MSCs. (**A**) FACS-CXCR4 staining. CXCR4 positive cells (primary murine MSCs) count from CD45^{neg}/CD11b^{neg}/Sca1^{pos}/CD140a^{pos} cells; control w/o CXCR4-antibody. Cells were fixated with 4% paraformaldehyde and permeabilized with 0.1% saponin (in DPBS/Gelafusal®). (**B**) CXCR4 immune fluorescence staining. Cells were fixated with methanol and permeabilized with DPBS/ Tween 20.

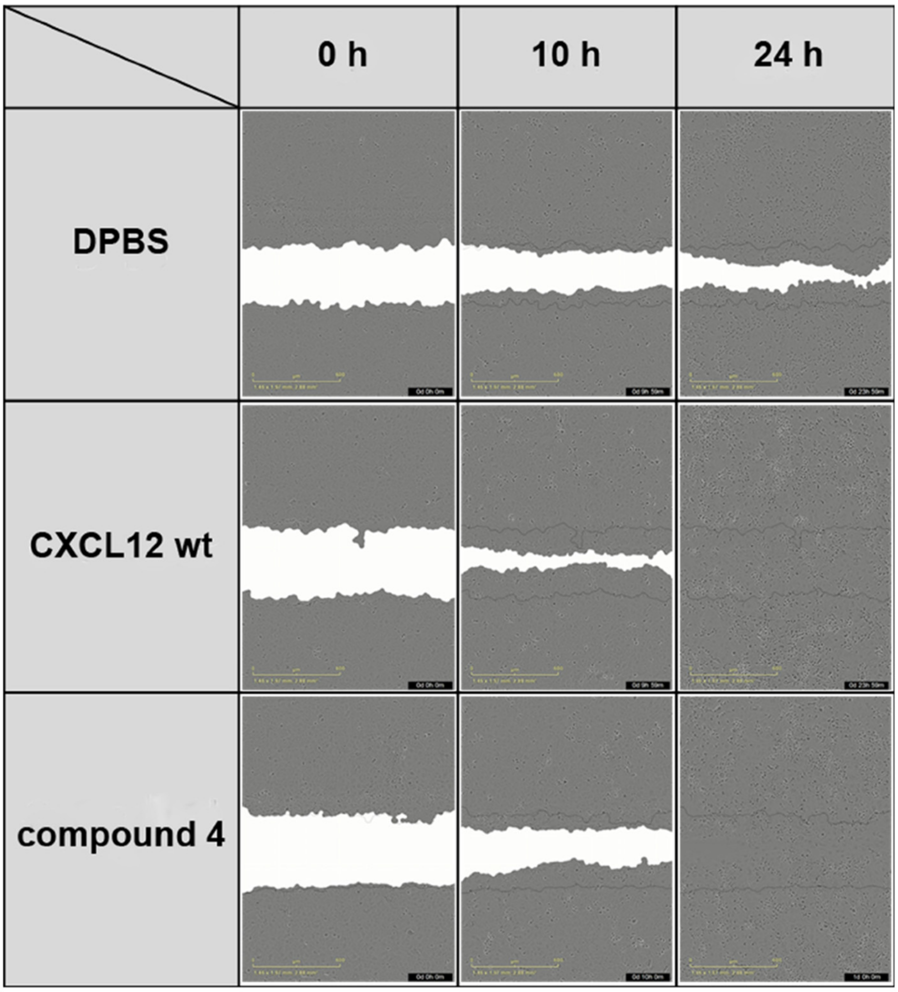

Figure S6. Representative pictures of gap closure after 0 h, 10 h, and 24 h by keratinocyte migration (HaCaT cells) in a scratch assay after pre-treatment with mitomycin C, a potent proliferation inhibitor. Cells were stimulated with 100 nM CXCL12 wt or compound 4.

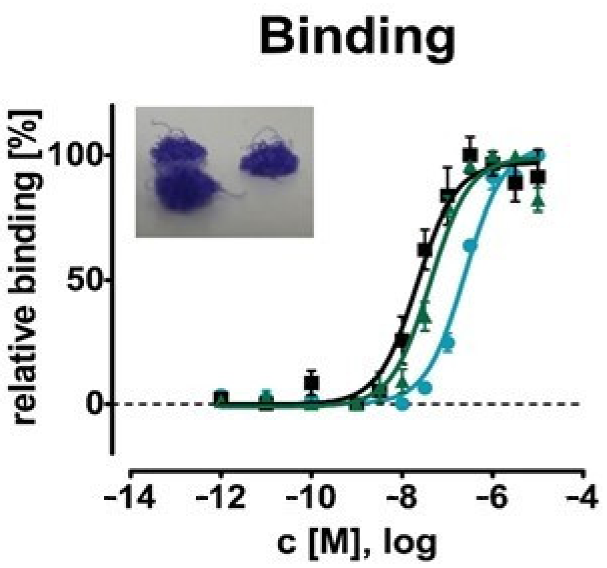

		EC_{50} [nM]	pEC_{50} (±SEM)
■	SBP	21.2	7.67 (±0.10)
▲	compound 1	41.5	7.38 (±0.08)
●	compound 2	240.0	6.62 (±0.05)

Figure S7. Binding affinities of indicated coatings shown on PCL-*co*-LC scaffolds in a concentration-dependent manner. Complete coating was achieved with 480 nM in case of compound 2. Data is presented as mean ± SEM, *n* = 3.

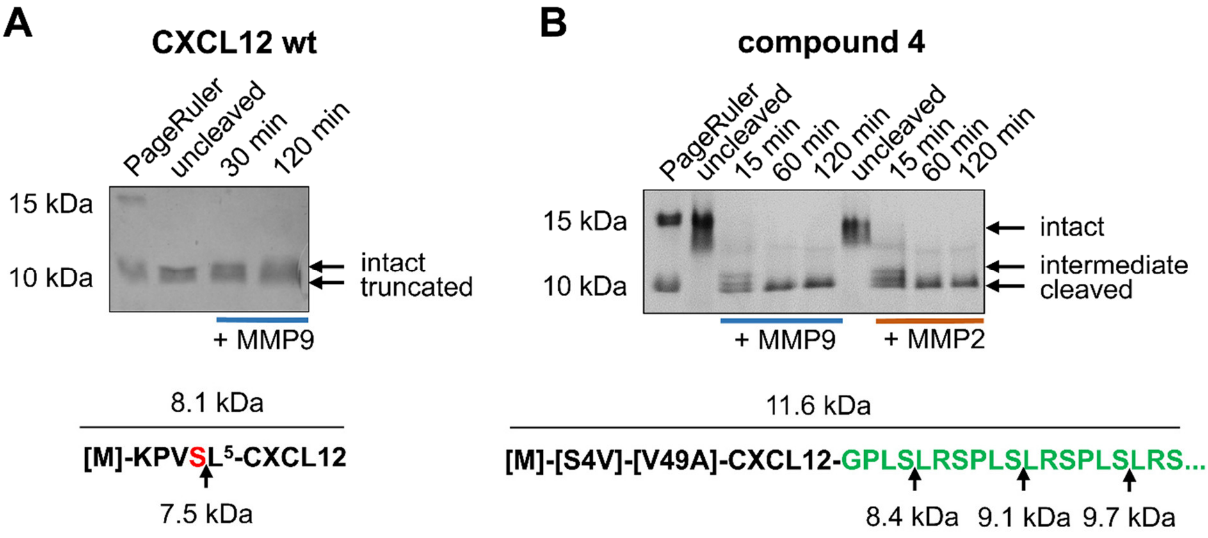

Figure S8. Stabilization and linker cleavage of modified CXCL12 in solution. (**A**) Truncation of native CXCL12 wt by MMP9 and (**B**) for the [S4V]-stabilized compound 4 with MMP9 and MMP2 (0.2 ng/µL), investigated by Tris/tricine SDS-PAGE. Corresponding cleavageproducts are indicated below.

A

human vs. porcine CXCL12

```
Range 1: 3 to 70  Graphics                                   Next Match  Previous Match

Score           Expect  Method                        Identities   Positives     Gaps
142 bits(357)   3e-51   Compositional matrix adjust.  66/68(97%)   68/68(100%)   0/68(0%)

1    --KPVSLSYRCPCRFFESHVARANVKHLKILNTPNCALQIVARLKNNNRQVCIDPKLKWIQEYLEKALNK----------   68
1    DEKPVSLSYRCPCRFFESHVARANIKHLKILNTPNCALQIVARLKSNNRQVCIDPKLKWIQEYLEKALNKPSPTLSGLTS   80

     -----------------
81   ALGSSLASTTVGITSRS   97
```

B

human vs. porcine CXCR4

```
Range 1: 1 to 337  Graphics                                    Next Match  Previous Match

Score            Expect  Method                        Identities     Positives      Gaps
655 bits(1691)   0.0     Compositional matrix adjust.  317/337(94%)   330/337(97%)   1/337(0%)

Query  1    MEGISIYTSDNYTEE-MGSGDYDSMKEPCFREENANFNKIFLPTIYSIIFLTGIVGNGLV  59
            M+G  I+TSDNYTE+ +GSGDYDS+KEPCFREENA+FN+IFLPT+YSIIFLTGIVGNGLV
Sbjct  1    MDGFRIFTSDNYTEDDLGSGDYDSIKEPCFREENAHFNRIFLPTVYSIIFLTGIVGNGLV  60

Query  60   ILVMGYQKKLRSMTDKYRLHLSVADLLFVITLPFWAVDAVANWYFGNFLCKAVHVIYTVN  119
            ILVMGYQKKLRSMTDKYRLHLSVADLLFV+TLPFWAVDAVANWYFG FLCKAVHVIYTVN
Sbjct  61   ILVMGYQKKLRSMTDKYRLHLSVADLLFVLTLPFWAVDAVANWYFGKFLCKAVHVIYTVN  120

Query  120  LYSSVLILAFISLDRYLAIVHATNSQRPRKLLAEKVVYVGVWIPALLLTIPDFIFANVSE  179
            LYSSVLILAFISLDRYLAIVHATNSQRPRKLLAEKVVYVGVWIPALLLTIPDFIFANV E
Sbjct  121  LYSSVLILAFISLDRYLAIVHATNSQRPRKLLAEKVVYVGVWIPALLLTIPDFIFANVRE  180

Query  180  ADDRYICDRFYPNDLWVVVFQFQHIMVGLILPGIVILSCYCIIISKLSHSKGHQKRKALK  239
             D RYICDRFYPNDLW+VVFQFQHIMVGLILPGIVILSCYCIIISKLSHSKG+QKRKALK
Sbjct  181  GDGRYICDRFYPNDLWLVVFQFQHIMVGLILPGIVILSCYCIIISKLSHSKGYQKRKALK  240

Query  240  TTVILILAFFACWLPYYIGISIDSFILLEIIKQGCEFENTVHKWISITEALAFFHCCLNP  299
            TTVILILAFFACWLPYYIGISIDSFILLEII+QGCEFE+TVHKWISITEALAFFHCCLNP
Sbjct  241  TTVILILAFFACWLPYYIGISIDSFILLEIIQQGCEFESTVHKWISITEALAFFHCCLNP  300

Query  300  ILYAFLGAKFKTSAQHALTSVSRGSSLKILSKGKRGG  336
            ILYAFLGAKFKTSAQHALTSVSRGSSLKILSKGKRGG
Sbjct  301  ILYAFLGAKFKTSAQHALTSVSRGSSLKILSKGKRGG  337
```

C

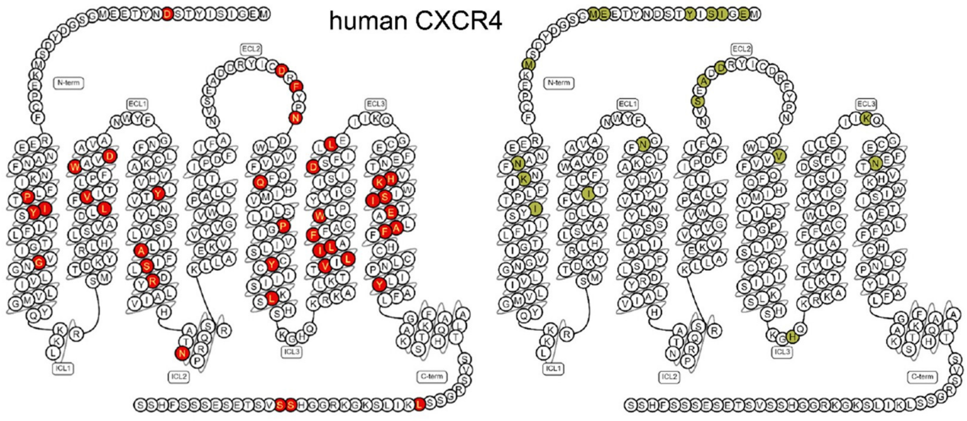

red = important residues for signaling

green = residues different in porcine receptor

Figure S9. Sequence alignment (NCBI BLAST) of human and porcine CXCL12 and CXCR4.(**A**) Human vs. porcine mature CXCL12 show 97% sequence identity. Differences where highlighted in red boxes. (**B**) Human CXCR4 isoform 1 precursor vs. porcine CXCR4 precursor display 94% sequence identity. (**C**) Snake plot (GPCRdb) of human CXCR4, left: red dots indicate important residues for CXCR4/CXCL12 signaling found by Wescott et al. Proc Natl Acad Sci USA 113 (2016) 9928-9933 [116]; right: green dots indicate residues which are different in porcine CXCR4. Only I44V in transmembrane helix 1 is overlapping important and different residue, but shares the same properties.
